# Premonitory urges are associated with decreased grey matter thickness within the insula and sensorimotor cortex in young people with Tourette syndrome

**DOI:** 10.1111/jnp.12089

**Published:** 2015-11-05

**Authors:** Amelia Draper, Georgina M. Jackson, Paul S. Morgan, Stephen R. Jackson

**Affiliations:** ^1^ School of PsychologyUniversity of NottinghamUK; ^2^ Institute of Mental HealthSchool of MedicineUniversity of NottinghamUK; ^3^ Medical Physics & Clinical EngineeringQueen's Medical CentreNottinghamUK

**Keywords:** Tourette syndrome, cortical thickness, premonitory urges, premonitory sensory phenomena, magnetic resonance imaging, insula, sensorimotor cortex

## Abstract

Tourette syndrome (TS) is a neurological disorder characterized by vocal and motor tics and is associated with cortical–striatal–thalamic–cortical circuit (CSTC) dysfunction and hyperexcitability of cortical limbic and motor regions, which are thought to lead to the occurrence of tics. Importantly, individuals with TS often report that their tics are preceded by ‘premonitory sensory phenomena’ (PSP) that are described as uncomfortable cognitive or bodily sensations that precede the execution of a tic, and are experienced as a strong urge for motor discharge. While the precise role played by PSP in the occurrence of tics is controversial, PSP are nonetheless of considerable theoretical and clinical importance in TS, not least because they form the core component in many of the behavioural therapies that are currently used in the treatment of tic disorders. In this study, we investigated the brain structure correlates of PSP. Specifically, we conducted a whole‐brain analysis of cortical (grey matter) thickness in 29 children and young adults with TS and investigated the association between grey matter thickness and PSP. We demonstrate for the first time that PSP are inversely associated with grey matter thickness measurements within the insula and sensorimotor cortex. We also demonstrate that grey matter thickness is significantly reduced in these areas in individuals with TS relative to a closely age‐ and gender‐matched group of typically developing individuals and that PSP ratings are significantly correlated with tic severity.

## Background

Tourette syndrome (TS) is a neurological disorder of childhood onset that is characterized by the presence of chronic vocal and motor tics (Cohen, Leckman, & Bloch, 2013). Tics are involuntary, repetitive, stereotyped behaviours that occur with a limited duration (Cohen *et al*., 2013). Motor tics can be simple or complex in appearance, ranging from repetitive movements to coordinated action sequences. Verbal tics can consist of repetitive sounds, words or utterances (palilalia), the production of inappropriate or obscene utterances (coprolalia), or the repetition of another's words (echolalia). Tics occur in bouts, typically many times in a single day, and are the most common form of movement disorder in children. TS is estimated to affect approximately 1% of individuals aged 5–18 years (Cohen *et al*., 2013).

Individuals with TS perceive a relatively constant demand to suppress their tics, particularly in social situations, and while the voluntary suppression of tics is possible in many cases, patients with TS typically report that it can be uncomfortable and stressful to suppress tics and that the urge to tic becomes uncontrollable after a period of suppression. Importantly, in the context of this study, individuals with TS report that their tics are often preceded by ‘premonitory sensory phenomena’ (PSP), sometimes referred to as premonitory urges, that are described as uncomfortable cognitive or bodily sensations that precede the execution of a tic, and are experienced as a strong urge for motor discharge (Bliss, 1980; Cohen *et al*., 2013; Leckman, Walker, & Cohen, 1993; Singer, 2005). PSP are most often measured using self‐report or questionnaire measurements, for example the Premonitory Urge for Tics Scale (PUTS; Woods, Piacentini, Himle, & Chang, 2005), and have been reported to be associated with tic severity scores (Ganos *et al*., 2015).

Understanding PSP is of theoretical and clinical importance. First, understanding PSP may lead to a different perspective on tics: in which the tics are viewed as a behavioural response that leads to the reduction of uncomfortable bodily sensations (Capriotti, Himle, & Woods, 2014) that are reinforced through the operation of dopamine (Buse, Schoenefeld, Münchau, & Roessner, 2013). Second, PSP have an important role in behavioural therapies that are currently used in the treatment of tic disorders: such as habit‐reversal therapy (Capriotti *et al*., 2014) and therapies based upon tic suppression (in which the child must learn to recognize the sensory events that often precede a tic, and use these to exert increased volitional control over motor and vocal outputs; Capriotti *et al*., 2014). In both of these examples, it is assumed that awareness of PSP can be used by an individual to enhance control over their tics.

Importantly, despite the theoretical and clinical importance of PSP, there is much that is still unclear about the nature of PSP, and there are good grounds for thinking that the occurrence of tics and the occurrence of PSP are independent. First, not all individuals with TS report experiencing PSP. In particular, children under 10 years of age, who present with simple tics, do not typically report being aware of PSP (Cohen *et al*., 2013; Leckman *et al*., 1993), and awareness of PSP increases with age across adolescence (Banaschewski, Woerner, & Rothenberger, 2003). This might indicate that the ability to recognize and articulate awareness of PSP is a consequence of general cognitive development, and perhaps the ability to distinguish self‐initiated volitional movements from involuntary movements, which emerges early during childhood. However, Banaschewski *et al*. (2003) note that awareness of premonitory sensations is not necessary for the successful suppression of tics, as 64% of their adolescent sample reported they were able to suppress their tics whereas only 37% reported premonitory sensations. As such, the occurrence of tics, and an individual's ability to suppress them, may occur independently of the awareness of PSP (Ganos *et al*., 2012).

Behavioural and functional brain imaging evidence indicates that PSP may be particularly associated with brain activity within the insular cortex (Bohlhalter *et al*., 2006; Ganos *et al*., 2015), which has been linked to interoceptive awareness (Craig, 2009). First, Ganos and colleagues investigated directly the relationship between the interoceptive awareness system and awareness of PSP in TS (Ganos *et al*., 2015). Using a heartbeat counting task as an index of interoceptive awareness, they reported that interoception was strongly associated with PSP – specifically, higher interoceptive awareness values were associated with increased PSP ratings. They also reported that increased tic severity scores were associated with increases in premonitory urges (Ganos *et al*., 2015).

Second, evidence for the involvement of the insular cortex in the perception of ‘urges‐for‐action’ more generally was provided by a quantitative meta‐analysis of functional brain imaging studies that had reported investigating the ‘urge‐for‐action’ associated with everyday behaviours such as yawning, swallowing, and micturition. This meta‐analytic study demonstrated that overlapping regions within the limbic sensory and motor regions – the insula and mid‐cingulate cortex – were common to all of these behaviours and overlapped spatially with regions that are strongly associated with the urge to tic in TS (Jackson, Parkinson, Kim, Schuermann, & Eickhoff, 2011).

Finally, it was recently reported that functional connectivity of the anterior insular cortex of the right hemisphere is associated both with the urge to tic in TS and with tic severity (Tinaz, Malone, Hallett, & Horovitz, 2015). Furthermore, a resting‐state functional magnetic resonance imaging (fMRI) study demonstrated that individuals with TS exhibited increased levels of functional connectivity involving the right dorsal anterior insula region and frontal–striatal brain areas that have been linked previously to the occurrence of tics in TS (Worbe *et al*., 2012, 2015). Importantly, Tinaz *et al*. (2015) demonstrated that functional connectivity between the right dorsal anterior insula region and bilateral supplementary motor area – an area associated with the cortical genesis of tics – was positively associated with the strength of PSP reported in the TS group.

In the current study, we conducted a whole‐brain analysis of cortical (grey matter) thickness in a sample of children and young adults with TS and investigated the relationship between grey matter thickness and PSP. We demonstrate for the first time that PSP in TS are inversely associated with grey matter thickness measurements within the insula cortex and sensorimotor cortex.

## Materials and methods

### Subjects

Thirty‐five children and adolescents with TS aged between 8 and 21 years were recruited from a specialized TS clinic, or through an advertisement on the *Tourettes Action* charity web page. However, following visual inspection of the data collected, six subjects were removed due to head movement artefact. Twenty‐nine children and adolescents with TS (3 females, mean age = 14 ± 3.1 years) were included in this study. All participants had a confirmed clinical diagnosis of TS. One participant had a diagnosis of comorbid attention‐deficit/hyperactivity disorder (ADHD), four participants had a diagnosis of comorbid autism spectrum disorder (ASD), and six participants had a diagnosis of comorbid obsessive‐compulsive disorder (OCD). On the day of testing, tic symptom severity was measured by administering the Yale Global Tic Severity Scale (YGTSS; Leckman *et al*., 1989) and premonitory urges were quantified by administering the PUTS (Woods *et al*., 2005). A group of 29 typically developing age‐ and gender‐matched adolescents (3 female, mean age = 14.3 ± 3.1 years) were used as a control group. Each control subject was born within 6 months of their matched subject with TS and was of the same gender. The Wechsler Abbreviated Scale of Intelligence (WASI) was used to measure IQ, and only subjects within a normal range of intelligence were included in the study. Further details of participants with TS can be seen in Table 1. Finally, a between‐groups *t*‐test confirmed there was no significant difference in IQ between the TS group and the matched controls (TS mean IQ = 110.4, control mean IQ = 116.8), *t*(56) = 1.8, *p* = .03. One subject with TS and one control subject were left‐handed.

**Table 1:** Details of participants with TS

ID	Gender	Age	WASI	YGSS	Motor	Phonic	PUTS	Comorbidity
TS01	M	18.0	103	20	11	9	3	–
TS02	M	19.1	111	70	22	18	16	–
TS03	M	15.4	118	3	3	0	14	–
TS04	M	14.0	123	63	23	20	21	OCD
TS05	M	16.8	118	19	9	0	16	–
TS06	M	10.0	96	10	6	4	16	ADHD
TS07	M	11.0	99	26	12	9	23	–
TS08	M	15.1	102	63	21	17	28	OCD
TS09	M	13.5	133	19	8	6	0	–
TS10	M	14.6	118	19	10	4	21	OCD
TS11	M	16.2	112	30	12	8	17	OCD
TS12	F	13.6	89	42	15	7	26	OCD
TS13	M	12.3	124	37	14	13	16	–
TS14	F	21.8	126	32	17	10	20	ASD
TS15	M	14.6	111	22	12	0	22	OCD
TS16	M	13.8	119	21	13	8	24	–
TS17	M	13.0	109	71	20	21	25	–
TS18	M	11.2	88	67	20	17	24	–
TS19	M	15.0	96	31	12	14	21	ASD
TS20	F	17.7	116	25	5	10	16	ASD
TS21	M	13.5	115	44	16	8	30	–
TS22	M	8.6	126	23	10	3	18	–
TS23	M	14.3	112	39	15	14	19	–
TS24	M	9.5	129	40	18	12	16	–
TS25	M	13.0	111	41	12	9	18	–
TS26	M	18.9	113	64	22	22	17	–
TS27	M	10.3	90	25	13	7	15	–
TS28	M	10.3	110	19	9	0	13	ASD
TS29	M	12.2	85	6	3	3	0	–

Note

WASI = Wechsler's abbreviated scale of intelligence: Matrix reasoning and vocabulary subtests. Motor and Phonic tic scores were measured using the Yale Global Tic Severity Scale on the day of testing. YGSS = Yale global severity score; PUTS = Premonitory Urge for Tics Scale; TS = Tourette syndrome; OCD = obsessive‐compulsive disorder; ADHD = attention‐deficit/hyperactivity disorder; ASD = autism spectrum disorder.

### Magnetic resonance imaging procedure

High‐resolution, T1‐weighted, anatomical images were acquired for all participants. Magnetization‐prepared rapid gradient‐echo (MPRAGE) scans were obtained using a Philips 3‐Tesla MRI scanner and a 32‐channel SENSE head coil. The MPRAGE scan consisted of 160 axial slices, with a 1 × 1 × 1 mm voxel size, and a field of view of 240 × 160 × 224 mm centred along the mid‐plane of the brain and angled to follow the AP–PC line using an initial survey scan. The TR was 8.26 ms. During this 4‐min scan, subjects were instructed to remain as still as possible. Foam padding was placed by the ears of the subject to restrict head movement.

### Cortical thickness data processing

Images were visually inspected and those with movement artefacts were removed from the data set. This included six subjects with TS and two control subjects, all of whom were under the age of thirteen. This leads to a sample size of 29 subjects with TS. We only included the control subjects that matched each subject with TS one‐for‐one, leading to 29 matched‐control subjects. Images were transformed into nifti format and then analysed using FreeSurfer software (http://surfer.nmr.mgh.harvard.edu/) to determine cortical thickness measures. FreeSurfer can be used to pre‐process the image data by correcting for motion, extracting brain tissue, transforming into standard Talairach space, and performing intensity normalization. The normalized images are segmented into anatomical structures (e.g., amygdala, hippocampus, caudate, ventricles, and putamen). Then, surface deformation is performed by following intensity gradients to closely estimate the position of the grey matter–white matter and the grey matter–cerebrospinal fluid boundaries, which are defined by identifying the location of the greatest shift in intensity (for more technical details on FreeSurfer software, please refer to the following publications; Dale, Fischl, & Sereno, 1999; Fischl, Sereno, & Dale, 1999). Grey matter thickness was measured as the closest distance between these two boundaries (Fischl & Dale, 2000).

## Results

### Association between grey matter thickness and PUTS ratings in the TS group

A general linear model (GLM) using QDEC GUI software (which is part of the FreeSurfer package) was used to investigate clusters of voxels where grey matter thickness values correlated significantly with PUTS scores in the TS group. An initial height threshold was set at *p* < .001, and identified clusters were then corrected for multiple comparisons (*p* < .05) and an extent threshold of 10 mm^2^ was applied. The results of this analysis are presented in Table 2.

**Table 2:** Regions where cortical grey matter thickness had a significant negative correlation with premonitory urge (Premonitory Urge for Tics Scale) scores

Cluster size (mm^2^)	Talairach coordinates			Label
	*x*	*y*	*z*	
67	60	−11	16	Right Rolandic Operculum
54	−29	−90	−2	Left Inferior Occipital Gyrus
15	−39	−17	−11	Left Insula
12	−33	−13	60	Left Pre‐Central Gyrus

Premonitory Urge for Tics Scale scores that were negatively correlated with grey matter thickness were identified in clusters located within sensorimotor cortical areas (S1, S2, and M1) and within the left insular cortex. By contrast, there were no significant clusters of positive correlations between PUTS scores and cortical grey matter thickness.

### Between‐group differences in cortical thickness

A GLM analysis was used to look for clusters of significant difference in grey matter thickness between groups. The statistical threshold was again set at *p* < .001, and clusters that were <10 mm^2^ were excluded. This analysis revealed there were many regions in which the TS group has significantly reduced GM thickness (cortical thinning) compared to the matched typically developing control group. These regions included the following: Bilateral sensorimotor and pre‐motor areas; bilateral insular cortex; and regions of pre‐frontal and parietal cortex. These data are described in full in a separate study (Draper, Jackson, Morgan & Jackson, 2015). However for the purposes of the current study, to aid brevity, and to maintain a clear focus on the anatomical correlates of premonitory urges in patients with TS, only those differences observed within sensorimotor and insular cortices (regions that were shown to be associated with PSP) will be reported here and considered further in this study. Relevant data are presented in Table 3. There were no areas in which GM thickness was significantly greater in the TS group. Note that each patient with TS was closely age‐ and gender‐matched with an individual typically developing control subject and the groups did not differ in IQ; thus, any between‐group effects due to age, gender, or IQ are assumed minimal and to be adequately controlled.

**Table 3:** Clusters where the Tourette syndrome group had significantly reduced GM thickness compared to the age‐ and gender‐matched typically developing control group

Region	Cluster size (mm^2^)	Talairach coordinates			Label
		*x*	*y*	*z*	
Sensorimotor cortex	366	−22	−11	54	Left precentral gyrus
	215	22	−22	67	Right precentral gyrus
	145	−39	5	13	Left precentral gyrus
	86	17	−27	39	Right paracentral lobule
	37	−55	−3	32	Left precentral gyrus
	34	−56	−18	33	Left postcentral gyrus
	28	5	−24	67	Right paracentral lobule
	17	−36	−34	45	Left postcentral gyrus
Insula and Cingulate cortex	640	−10	−21	38	Left posterior cingulate
	183	−3	27	−2	Left rostral anterior cingulate
	101	35	−1	14	Right insula
	64	−29	19	8	Left insula
	55	7	8	37	Right caudal anterior cingulate
	26	−14	40	10	Left rostral anterior cingulate
	11	11	−34	39	Right posterior cingulate
	10	−8	−52	26	Left isthmus

### Relationship between PUTS ratings, tic severity scores, IQ, and age

**Figure 1:**
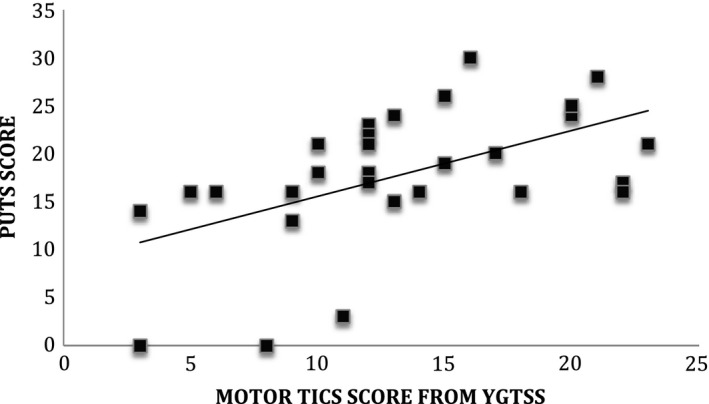
Scatter plot illustrating the positive association between motor tic severity scores from the Yale Global Tic Severity Scale (YGTSS) and premonitory urges (Premonitory Urge for Tics Scale [PUTS] scores).

Previous studies have reported that PSP (as measured by the PUTS) are associated with tic severity (Ganos *et al*., 2012). For this reason, we investigated the relationship between premonitory urges (i.e., PUTS ratings), motor and phonic tic severity, IQ, and age. As predicted, a positive correlation was found between premonitory urges, as measured by the PUTS, and motor tic severity (Pearson's *R* = .53, *p* < .002; Figure 1) and, to a lesser extent, phonic tic severity (Pearson's *R* = .32, *p* < .05) as measured by the YGTSS. By contrast, there was no significant relationship between age and PUTS scores (Pearson's *R* = −.03, *p* > .1), or IQ and PUTS scores (Pearson's *R* = −.06, *p* > .1).

## Discussion

Premonitory urges, or PSP, are of considerable theoretical and clinical importance in understanding and treating TS and tic disorders, and yet their precise role in the occurrence of tics, and their relationship to tics, remains controversial and the subject of much debate. In the current study, we conducted a whole‐brain analysis of cortical (grey matter) thickness in a sample of children and young adults with TS and investigated the relationship between grey matter thickness and PSP. We demonstrated for the first time that PSP reported by individuals with TS are inversely associated with grey matter thickness measurements within the right sensorimotor cortex and the left insula. We also demonstrated that cortical thickness within the sensorimotor cortex, insula, and anterior cingulate cortex was significantly reduced within the TS group relative to a closely age‐ and gender‐matched group of typically developing young adults. Finally, we replicated a previous finding that PSP are significantly correlated with phonic and motor tic severity. By contrast, we did not replicate a previous report demonstrating that PSP increase with age. These findings are discussed below.

### Role of the insula in the urge to tic

In the current study, we demonstrated that cortical thickness within the insular and sensorimotor cortex was inversely associated with ratings of the strength of premonitory urges in young adults with TS and that cortical thickness within the sensorimotor cortex, anterior cingulate, and insula was significantly reduced relative to a matched group of typically developing controls. Our finding of significant cortical thinning within the sensorimotor, insular, and cingulate cortices of individuals with TS confirms similar findings reported by a number of other research groups (Draganski *et al*., 2010; Fahim *et al*., 2010; Sowell *et al*., 2008; Worbe *et al*., 2010).

Our demonstration that cortical thickness within the insular and sensorimotor cortex was inversely associated with ratings of the strength of premonitory urges in young adults with TS supports the view, expressed previously, that the insula and cingulate cortex may be the most likely anatomical regions responsible for the uncomfortable feelings associated with the premonitory urge to tic (Bohlhalter *et al*., 2006). Consistent with this proposal, a previous fMRI study has reported that the insula and operculum were amongst a network of brain regions that increased their activity immediately prior to the occurrence of tics in TS (Bohlhalter *et al*., 2006), which is also with the demonstration that electrical stimulation of the insular cortex, or the parietal operculum, elicits unpleasant somatosensory or visceral sensations (Augustine, 1996; Ostrowsky *et al*., 2002; Penfield & Faulk, 1955). This proposal is also consistent with the putative central role for these areas in the neural representation of bodily states more generally, and with the initiation of behaviours associated with such bodily representations (for reviews, see Craig, 2002, 2009). Based upon a meta‐analytic analysis of fMRI studies of behavioural urges (Jackson *et al*., 2011), it was proposed that the insular and opercular regions, together with the cingulate motor cortex, may be central in representing the urge‐for‐action, and together these regions form a neural circuit that represents bodily sensations, generates an urge‐for‐action, selects a particular action based upon a cost–benefit analysis of the likely ‘value’ of that action, accumulates evidence on the outcomes of that action, determines whether the conditions giving rise to the urge have been resolved, and, if appropriate, generates a sense that the urge has been satisfied (Jackson *et al*., 2011).

A core theoretical issue, which is also of considerable clinical importance given their central role in behavioural therapies, has been whether PSP represent abnormally activated bodily or interoceptive states, as some have suggested (Capriotti *et al*., 2014), or whether they arise simply as a consequence of suppressing or withholding tics, and are therefore not different to the uncomfortable bodily sensations that often accompany other ‘urges‐for‐action’ in which actions (e.g., micturition, yawning, and sneezing) are withheld or delayed (Jackson *et al*., 2011). While the results of functional imaging studies can be interpreted as supporting the notion of abnormally activated bodily or interoceptive states in TS, an important caveat for such studies is that participants are required to remain still while being scanned. This may be more difficult in individuals who may need to suppress their tics, and remaining still and suppressing tics may require substantial additional self‐monitoring in the TS group. Each of these factors might conceivably lead to localized increases in fMRI BOLD signal, or functional connectivity, in patients with TS relative to controls, that need not support the conclusion that individuals with TS exhibit abnormally activated bodily or interoceptive states. For this reason, demonstrations that measures of brain anatomy within the insula and sensorimotor cortex are significantly altered in individuals with TS and that variation in these same measures of brain anatomy significantly predict individual levels of PSP strike us as more compelling evidence for abnormal bodily or interoceptive states in TS. In this context, it is of interest to note that a recent positron emission tomography (PET) imaging study has revealed that there are widespread alterations in GABA_A_ receptor binding in individuals with TS that include the insular cortex (Lerner *et al*., 2012) and a post‐mortem study has indicated that there may be reduced numbers of GABAergic interneurons within the insula (Vaccarino, Kataoka, & Lennington, 2013). Both of these factors would highly likely to contribute significant changes in the inhibitory–excitatory balance within the insula.

In the current study, we replicated the finding that PSP ratings are significantly correlated with tic severity scores. This finding adds confidence to the claim that tic severity and PSP are often strongly associated.

### Summary

Individuals with TS report that their tics are often preceded by PSP or premonitory urges that are described as uncomfortable bodily sensations that precede the execution of a tic and experienced as a strong urge for motor discharge. Understanding the physiological and anatomical basis for such ‘urges‐for‐action’ is theoretically important as it may inform our understanding of those mechanisms that govern impulse control more generally, and is of particular clinical importance in TS insofar as behavioural therapies that are currently used in the treatment of tic disorders rely to a large extent on an individual's ability to perceive and use PSP to control their tics. In the current study, we conducted a whole‐brain analysis of cortical (grey matter) thickness in a sample of children and young adults with TS and investigated the relationship between grey matter thickness and PSP. We demonstrate for the first time that premonitory urges in TS are inversely associated with grey matter thickness measurements within the sensorimotor and insular cortices and that cortical thickness within the sensorimotor cortex, insula, and anterior cingulate cortex is significantly reduced within the TS group relative to a closely age‐ and gender‐matched group of typically developing young adults. Finally, we replicated a previous finding that PSP are significantly correlated with phonic and motor tic severity.

## References

[jnp12089-bib-0001] Augustine, J. R. (1996). Circuitry and functional aspects of the insular lobe in primates including humans. Brain research. Brain Research Reviews, 22, 229–244. doi:10.1016/S0165‐0173(96)00011‐2 8957561 10.1016/s0165-0173(96)00011-2

[jnp12089-bib-0002] Banaschewski, T. , Woerner, W. , & Rothenberger, A. (2003). Premonitory sensory phenomena and suppressibility of tics in Tourette syndrome: Developmental aspects in children and adolescents. Developmental Medicine and Child Neurology, 45, 700–703. doi:10.1111/j.1469‐8749.2003.tb00873.x 14515942 10.1017/s0012162203001294

[jnp12089-bib-0003] Bliss, J. (1980). Sensory experiences of Gilles de la Tourette syndrome. Archives of General Psychiatry, 37, 1343–1347. doi:10.1001/archpsyc.1980.01780250029002 6934713 10.1001/archpsyc.1980.01780250029002

[jnp12089-bib-0004] Bohlhalter, S. , Goldfine, A. , Matteson, S. , Garraux, G. , Hanakawa, T. , Kansaku, K. , Wurzman, R. , & Hallett, M. (2006). Neural correlates of tic generation in Tourette syndrome: An event‐related functional MRI study. Brain, 129, 2029–2037. doi:10.1093/brain/awl050 16520330 10.1093/brain/awl050

[jnp12089-bib-0005] Buse, J. , Schoenefeld, K. , Münchau, A. , & Roessner, V. (2013). Neuromodulation in Tourette syndrome: Dopamine and beyond. Neuroscience and Biobehavioral Reviews, 37, 1069–1084. doi:10.1016/j.neubiorev.2012.10.004 23085211 10.1016/j.neubiorev.2012.10.004

[jnp12089-bib-0006] Capriotti, M. R. , Himle, M. B. , & Woods, D. W. (2014). Behavioral treatments for Tourette syndrome. Journal of Obsessive‐Compulsive and Related Disorders, 3, 415–420. doi:10.1016/j.jocrd.2014.03.007 10.1016/j.jocrd.2014.03.007 PMC6150491 30245958

[jnp12089-bib-0007] Cohen, S. C. , Leckman, J. F. , & Bloch, M. H. (2013). Clinical assessment of Tourette syndrome and tic disorders. Neuroscience and Biobehavioral Reviews, 37, 997–1007. doi:10.1016/j.neubiorev.2012.11.013 23206664 10.1016/j.neubiorev.2012.11.013 PMC3674220

[jnp12089-bib-0008] Craig, A. D. (2002). How do you feel? Interoception: The sense of the physiological condition of the body. Nature Reviews Neuroscience, 3, 655–666. doi:10.1038/nrn894 12154366 10.1038/nrn894

[jnp12089-bib-0009] Craig, A. D. (2009). How do you feel – now? The anterior insula and human awareness. Nature Reviews Neuroscience, 10, 59–70. doi:10.1038/nrn2555 10.1038/nrn2555 19096369

[jnp12089-bib-0010] Dale, A. M. , Fischl, B. , & Sereno, M. I. (1999). Cortical surface‐based analysis. I. Segmentation and surface reconstruction. NeuroImage, 9, 179–194. doi:10.1006/nimg.1998.0395 9931268 10.1006/nimg.1998.0395

[jnp12089-bib-0011] Draganski, B. , Martino, D. , Cavanna, A. E. , Hutton, C. , Orth, M. , Robertson, M. M. , … Frackowiak, R. S. (2010). Multispectral brain morphometry in Tourette syndrome persisting into adulthood. Brain, 133, 3661–3675. doi:10.1093/brain/awq300 21071387 10.1093/brain/awq300 PMC2995885

[jnp12089-bib-0101] Draper, A. , Jackson, G. M. , Morgan, P. S. , & Jackson, S. R. (2015). Alterations in cortical grey matter thickness in young people with Tourette syndrome. Manuscript in preparation.

[jnp12089-bib-0012] Fahim, C. , Yoon, U. , Das, S. , Lyttelton, O. , Chen, J. , Arnaoutelis, R. , Evans, A. C. (2010). Somatosensory‐motor bodily representation cortical thinning in Tourette: Effects of tic severity, age and gender. Cortex, 46, 750–760. doi:10.1016/j.cortex.2009.06.008 19733347 10.1016/j.cortex.2009.06.008

[jnp12089-bib-0013] Fischl, B. , & Dale, A. M. (2000). Measuring the thickness of the human cerebral cortex from magnetic resonance images. Proceedings of the National Academy of Sciences of the United States of America, 97, 11050–11055. doi:10.1073/pnas.200033797 10984517 10.1073/pnas.200033797 PMC27146

[jnp12089-bib-0014] Fischl, B. , Sereno, M. I. , & Dale, A. M. (1999). Cortical surface‐based analysis. II: Inflation, flattening, and a surface‐based coordinate system. NeuroImage, 9, 195–207. doi:10.1006/nimg.1998.0396 9931269 10.1006/nimg.1998.0396

[jnp12089-bib-0015] Ganos, C. , Garrido, A. , Navalpotro‐Gómez, I. , Ricciardi, L. , Martino, D. , Edwards, M. J. , , , … Bhatia, K. P. (2015). Premonitory urge to tic in Tourette's is associated with interoceptive awareness. Movement Disorders. 30, 1198–1202 doi:10.1002/mds.26228 25879819 10.1002/mds.26228

[jnp12089-bib-0016] Ganos, C. , Kahl, U. , Schunke, O. , Kuhn, S. , Haggard, P. , Gerloff, C. , … Münchau, A. (2012). Are premonitory urges a prerequisite of tic inhibition in Gilles de la Tourette syndrome? Journal of Neurology, Neurosurgery, and Psychiatry, 83, 975–978. doi:10.1136/jnnp‐2012‐303033 10.1136/jnnp-2012-303033 22842713

[jnp12089-bib-0017] Jackson, S. R. , Parkinson, A. , Kim, S. Y. , Schuermann, M. , & Eickhoff, S. B. (2011). On the functional anatomy of the urge‐for‐action. Cognitive Neuroscience, 3–4, 252–257. doi:10.1080/17588928.2011.604717 10.1080/17588928.2011.604717 PMC3259619 22299020

[jnp12089-bib-0018] Leckman, J. F. , Riddle, M. A. , Hardin, M. T. , Ort, S. I. , Swartz, K. L. , Stevenson, J. , & Cohen, D. J. (1989). The Yale Global Tic Severity Scale: Initial testing of a clinician‐rated scale of tic severity. Journal of the American Academy of Child and Adolescent Psychiatry, 28, 28566–28573. doi:10.1097/00004583‐198907000‐00015 10.1097/00004583-198907000-00015 2768151

[jnp12089-bib-0019] Leckman, J. F. , Walker, D. E. , & Cohen, D. J. (1993). Premonitory urges in Tourette's syndrome. American Journal of Psychiatry, 150, 98–102. doi:10.1176/ajp.150.1.98 8417589 10.1176/ajp.150.1.98

[jnp12089-bib-0020] Lerner, A. , Bagic, A. , Simmons, J. M. , Mari, Z. , Bonne, O. , Xu, B. , … Hallett, M. (2012). Widespread abnormality of the g‐aminobutyric acid‐ergic system in Tourette syndrome. Brain, 135, 1926–1936. doi:10.1093/brain/aws104 22577221 10.1093/brain/aws104 PMC3359755

[jnp12089-bib-0021] Ostrowsky, K. , Magnin, M. , Ryvlin, P. , Isnard, J. , Guenot, M. , & Mauguiere, F. (2002). Representation of pain and somatic sensation in the human insula: A study of responses to direct electrical cortical stimulation. Cerebral Cortex, 12, 376–385. doi:10.1093/cercor/12.4.376 11884353 10.1093/cercor/12.4.376

[jnp12089-bib-0022] Penfield, W. , & Faulk, M. E. (1955). The insula: Further observations on its function. Brain, 78, 445–470. doi:10.1093/brain/78.4.445 13293263 10.1093/brain/78.4.445

[jnp12089-bib-0023] Singer, H. S. (2005). Tourette's syndrome: From behaviour to biology. The Lancet Neurology, 4, 149–159. doi:10.1016/S1474‐4422(05)01012‐4 15721825 10.1016/S1474-4422(05)01012-4

[jnp12089-bib-0024] Sowell, E. R. , Kan, E. , Yoshii, J. , Thompson, P. M. , Bansal, R. , Xu, D. , … Peterson, B. S. (2008). Thinning of sensorimotor cortices in children with Tourette syndrome. Nature Neuroscience, 11, 637–639. doi:10.1038/nn.2121 18488025 10.1038/nn.2121 PMC2605107

[jnp12089-bib-0025] Tinaz, S. , Malone, P. , Hallett, M. , & Horovitz, S. G. (2015). Role of the right dorsal anterior insula in the urge to tic in Tourette syndrome. Movement Disorders, 30, 1190–1197. doi:10.1002/mds.26230 25855089 10.1002/mds.26230 PMC5088605

[jnp12089-bib-0026] Vaccarino, F. M. , Kataoka, Y. , & Lennington, J. (2013). Cellular and molecular pathology in Tourette syndrome In Martino D. & Leckman J. F. (Eds.), Tourette syndrome (pp. 205–220). Oxford, UK: Oxford University Press.

[jnp12089-bib-0027] Woods, D. W. , Piacentini, J. , Himle, M. B. , & Chang, S. (2005). Premonitory Urge for Tics Scale (PUTS): Initial psychometric results and examination of the premonitory urge phenomenon in youths with Tic disorders. Journal of Developmental and Behavioral Pediatrics, 26, 397–403. doi:10.1097/00004703‐200512000‐00001 16344654 10.1097/00004703-200512000-00001

[jnp12089-bib-0028] Worbe, Y. , Gerardin, E. , Hartmann, A. , Valabregue, R. , Chupin, M. , Tremblay, L. , … Lehéricy, S. (2010). Distinct structural changes underpin clinical phenotypes in patients with Gilles de la Tourette syndrome. Brain, 133, 3649–3660. doi:10.1093/brain/awq293 20959309 10.1093/brain/awq293

[jnp12089-bib-0029] Worbe, Y. , Malherbe, C. , Hartmann, A. , Pelegrini‐Issac, M. , Messe, A. , Vidailhet, M. , … Benali, H. (2012). Functional immaturity of cortico‐basal ganglia networks in Gilles de la Tourette syndrome. Brain, 135, 1937–1946. doi:10.1093/brain/aws056 22434213 10.1093/brain/aws056

[jnp12089-bib-0030] Worbe, Y. , Marrakchi‐Kacem, L. , Lecomte, S. , Valabregue, R. , Poupon, F. , Guevara, P. , … Poupon, C. (2015). Altered structural connectivity of cortico–striato–pallido–thalamic networks in Gilles de la Tourette syndrome. Brain, 138, 472–482. doi:10.1093/brain/awu311 25392196 10.1093/brain/awu311 PMC4306818

